# COMPARING EXERCISE DETERMINANTS BETWEEN BLACK AND WHITE OLDER ADULTS WITH HEART FAILURE

**DOI:** 10.1093/geroni/igac059.983

**Published:** 2022-12-20

**Authors:** Navin Kaushal, Donya Nemati, Dylan Mann-Krzisnik, Adrian Noriega de la Colina

**Affiliations:** Indiana University, Indianapolis, Indiana, United States; Indiana University, Indianapolis, Indiana, United States; McGill University, Montreal, Quebec, Canada; McGill University, Westmount, Quebec, Canada

## Abstract

Heart Failure (HF) is a leading cause of mortality among older-adults (OA); however, engaging in moderate-to-vigorous intensity of physical activity (MVPA) improves survival rates. Limited research has applied behavior theory to understand determinants of MVPA among OA with HF and compared the effects between races. This study aimed to use the Health Belief Model (HBM) to compare MVPA determinants between Black and White OA diagnosed with HF.

Methods: The HF-ACTION Trial is a multi-site trial that helped facilitate physical activity among individuals with HF. The present study used structural equation modeling to test the change in HBM determinants and MVPA across 12 months among OA.

Results: Participants were OA 72.28 (SD= 5.41) years old, (Black: n= 230; White, n= 441). The model found MVPA to be facilitated by perceived benefits (Black: β=.27, p=.001; White: β=.28; p<.001), self-efficacy (Black: β=.20, p=.003; White: β=.24; p<.001) and deterred by perceived barriers (Black: β=-.25, p=.001; White β=-.24; p<.001). Perceived threats deterred Black OA (β=-.23; p=.002) but not White OA (β=-.07; p=.278) from MVPA. Experimental arm predicted the development of self-efficacy (Black: β=.21. p=.001; White: β=.23; p=.001), and perceived benefits (Black: β=.21. p=.001; White: β=.23; p=.001) but not barriers (p>.05) for both races.

Conclusions: Perceived barriers was a strong determinant to MVPA, and it was not resolved in the experimental arm for either race. The experimental arm did not resolve perceived threat, which was a deterrent for Black OA. Individual characteristics need to be considered when designing programs for HF patients to reduce health disparity.

